# Liquid Biopsy-Based DNA Methylation Biomarkers for Precision Medicine in Breast Cancer

**DOI:** 10.1017/erm.2025.10008

**Published:** 2025-06-17

**Authors:** Ieva Sadzeviciene, Danielius Kaubrys, Sonata Jarmalaite

**Affiliations:** 1 Institute of Biosciences, Life Sciences Center, Vilnius University, Vilnius, Lithuania; 2 National Cancer Institute, Biobank, Vilnius, Lithuania

**Keywords:** breast cancer, cell-free DNA (cfDNA), DNA hypermethylation, DNA methylation biomarkers, genome-wide methylation, liquid biopsy

## Abstract

**Background:**

Current breast cancer (BC) diagnostics include detailed pathological and genetic analysis for biological subtype identification; however, throughout the course of the disease, new alterations determining the progression of the disease or resistance to treatment appear. The tests based on liquid biopsy allow minimally invasive real-time monitoring of tumour-specific alteration during the entire disease treatment. Tumour-specific genetic material fragments occur in bodily fluids, and cell-free nucleic acids are a convenient tool for analysing genetic and epigenetic changes in tumours. Evidence for the diagnostic and prognostic value of epigenetic biomarkers is gradually increasing. Although, up to date, there is limited access to *in vitro* diagnostic (IVD) epigenetic liquid biopsy-based tests for BC management, the data on the clinical potential of such tests and biomarkers are accumulating rapidly.

**Methods:**

In this review, we focused on research involving cell-free DNA methylation biomarkers in blood serum or plasma samples from BC patients.

**Results:**

Our review systematises data from genome-wide and targeted studies of DNA methylation changes in liquid biopsies from BC patients, aiming to highlight the most critical biomarkers suitable for early BC diagnosis, treatment personalisation and prognosis.

**Conclusion:**

In summary, cell-free DNA methylation biomarkers show strong potential to enhance breast cancer diagnosis, prognosis, and personalised treatment through integrated clinical profiling.

## Introduction

Breast cancer (BC) is one of the most common malignancies and the most diagnosed neoplasm among women globally (Ref. 1). The 5-year survival rate for BC varies widely depending on the stage of the disease at diagnosis. The estimated 5-year survival for patients with BC diagnosed at Stages I and II ranges from 92% to 100% and decreases drastically to 74% at Stage III and 23% at Stage IV (Ref. 2). The substantial increase in mortality as cancer progresses suggests the importance of early-stage diagnosis and personalised disease management, which has the potential to increase survival rates significantly.

BC diagnostics often involve multiple techniques, such as mammography, magnetic resonance imaging and ultrasound. While these methods are widely applied, they have limitations, such as a high probability of false positives or negatives (Ref. 3). Additionally, the gold standard for BC diagnosis is tumour tissue biopsy; however, this technique is invasive, can cause a risk of infection and can yield inconclusive results due to tumour heterogeneity and potential sample size inadequacy (Ref. 3).

**Figure 1.**
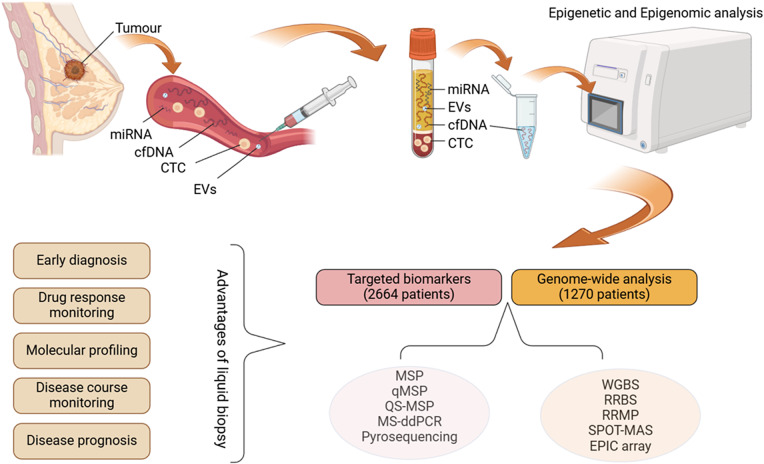
Liquid biopsy workflow and the methods used in reviewed articles (Created with BioRender.com). cfDNA: cell-free DNA; CTC: circulating tumour cell; EV: extracellular vesicles; miRNA: microRNA; MSP: methylation-specific PCR; qMSP: quantitative methylation-specific PCR; OS-MSP: one-step methylation-specific PCR; MS-ddPCR: methylation-specific droplet digital PCR; WGBS: whole-genome bisulphite sequencing; RRBS: reduced representation bisulphite sequencing; RRMP: reduced representative methylome profiling; SPOT-MAS: screening for the presence of tumours by DNA methylation and size.

Liquid biopsy has emerged as a credible alternative to traditional diagnostic methods, offering numerous benefits. It involves the collection of a sample of blood followed by the analysis of its components, which include circulating tumour cells (CTCs), cell-free/circulating tumour DNA (cf/ctDNA), exosomes, microRNA (miRNA) and proteins (Refs 4, 5, 6, 7). The technique’s clinical benefits include its minimally invasive nature, the ability to perform serial sampling and the ability to generate a systemic picture of (epi)genomic changes in various tumour foci and metastases. This method allows real-time monitoring of tumour progression dynamics and potentially informs on the presence of minimal residual disease (Figure 1) (Ref. 8).

Most of the research on liquid biopsies has focused on differentiating gene mutational profiles between cancer patients and healthy controls. However, liquid biopsy is also a valuable tool for analysing epigenetic changes. Epigenetic changes include DNA and histone modifications as well as non-coding RNAs (ncRNA). One of the major epigenetic alterations involves DNA methylation – a widespread modification in which a methyl group is added to CpG dinucleotides (Ref. 9). DNA methylation is an essential process in regulating gene transcription and neoplasm formation. Hypermethylation of specific loci, such as tumour suppressors, has been widely observed in cancer tissues, leading to the downregulation of the expression of these genes (Ref. 10). However, global genomic hypomethylation has also been recognised as a key driver of tumorigenesis, leading to mobile genomic elements and oncogene activation, thus accelerating tumour progression and metastatic lesion formation (Ref. 11). These methylation patterns can often be detected through liquid biopsy by analysing cell-free DNA (cfDNA), which shows promise in facilitating early diagnosis and predicting tumour response to therapy (Ref. 9).

Since 1999, when Wong et al. and Esteller et al. discovered cancer-related aberrant DNA methylation in patients’ serum, the importance of DNA methylation research in liquid biopsies has markedly increased, and the list of biomarkers is constantly growing (Refs 12, 13). Examples of such cfDNA biomarkers for BC are the *ESR1* and *SFN* genes, whose promoters are hypermethylated in BC and enable credible differentiation of BC patients from healthy controls (Ref. 14). High *PTEN* methylation has been associated with the late stages of BC, suggesting that *PTEN* methylation is a prognostic biomarker (Ref. 15). Additionally, aberrant methylation of *TMEM240* has been correlated with poor response to hormone therapy in BC patients (Ref. 16). The results of such studies highlight the potential of liquid biopsy for improving BC diagnostics and prognostics and predicting patient response to treatment. Moreover, DNA methylation is a stable and easily detectable change, allowing us to analyse it in tumour genetic material and liquid biopsy (Ref. 17). On the other hand, epigenetic profiling could provide several benefits to overcome limitations associated with mutation-based liquid biopsy analysis. For example, genetic mutations in cancer can be rare and difficult to predict, whereas DNA methylation changes can be more abundant and more accessible to detect (Ref. 18).

Similarly to DNA methylation changes, variations in certain ncRNA molecules, such as miRNA, long non-coding RNA and circular RNA, have been shown to have both diagnostic and prognostic potential in BC (Refs 19, 20, 21, 22). Biomarkers such as miR-21, LINC00511 and hsa_circ_0001785 were associated with BC progression and metastasis (Refs 19, 20, 21). Moreover, several miRNAs were upregulated in triple-negative breast cancer (TNBC) and correlated with poor survival, thus supporting the importance of ncRNA analysis alongside cfDNA studies (Ref. 22).

In liquid biopsies, simple PCR-based methods allow fast and reliable biomarker detection, circumventing the need for time-consuming and expensive genomic analysis of cfDNA. However, based on the universal nature of epigenetic changes, tumour type specificity of such biomarkers can be quite low. Further efforts are needed to identify BC-specific, informative and reliable sets of biomarkers suited for clinical application.

This article reviews the existing research on DNA methylation biomarkers in BC obtained through liquid biopsy by providing specific examples, biomarker categories and potential in BC diagnosis, prognosis and prediction of treatment responses.

## Methods

**Figure 2.**
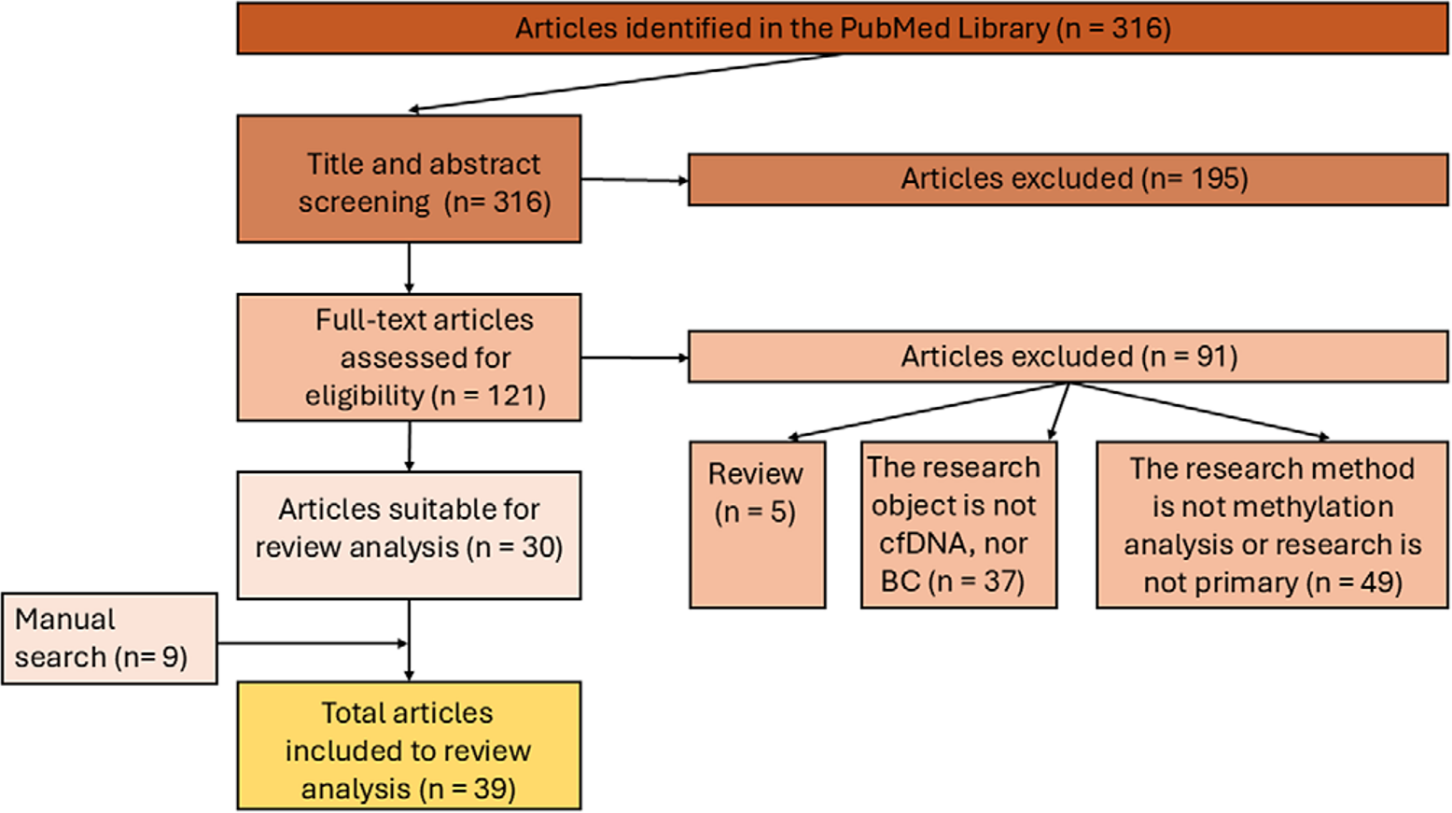
The inclusion/exclusion chart and search method related to the review.

Although interest in BC-specific cfDNA methylation analysis has increased more than twofold since 2016, the search for publications was performed on the PubMed database (https://pubmed.ncbi.nlm.nih.gov/) without time limits, covering the period from inception (1998) to 20 April 2024. The following descriptors were used for database searches: (“breast neoplasms”[MeSH Terms] OR (“breast”[All Fields] AND “neoplasms”[All Fields]) OR “breast neoplasms”[All Fields] OR (“breast”[All Fields] AND “cancer”[All Fields]) OR “breast cancer”[All Fields]) AND (“cell free nucleic acids”[MeSH Terms] OR (“cell free”[All Fields] AND “nucleic”[All Fields] AND “acids”[All Fields]) OR “cell free nucleic acids”[All Fields] OR (“cell”[All Fields] AND “free”[All Fields] AND “dna”[All Fields]) OR “cell free dna”[All Fields]) AND (“dna methylation”[MeSH Terms] OR (“dna”[All Fields] AND “methylation”[All Fields]) OR “dna methylation”[All Fields]). The literature search generated 316 results. Appropriate studies were selected through a two-step publication analysis: screening by title and abstract, and full-text analysis leading to the final 39 publications suitable for review analysis (Figure 2).

### Inclusion criteria

BC cfDNA methylation analysis in liquid biopsies (blood, plasma and serum) and the English language were the primary selection criteria for the papers. The dataset used in this review comprised demographic data from the study group, BC subtypes, analysis techniques, methylated biomarker sensitivity (the proportion of true positive cases in the analysed BC patients’ cohort) and specificity (the proportion of true negative cases in the cohort of non-cancerous samples, and diagnostic or prognostic values) (Supplementary Tables S1 and S2).

### Exclusion criteria

Excluded articles were either irrelevant to the topic or study objective; incomplete descriptions of the methods or study groups were also reasons for exclusion. All reviews, letters, case studies, conference material and cohort analyses performed from non-primary studies were excluded from the present analysis.

Considering the selection criteria, 31 articles on target biomarkers and 8 articles analysing the methylome were used for further analysis.

### Analysis of the identified biomarkers using the DAVID bioinformatics tool

The Database for Annotation, Visualization, and Integrated Discovery (DAVID; https://davidbioinformatics.nih.gov/) was performed on the analysed biomarker sets (Refs 23, 24). The Gene–Disease Association Database (GAD) was employed to categorise the biomarkers according to their known involvement in human disease categories. The functional annotation analysis associated methylated biomarkers with various biological processes. Furthermore, the Kyoto Encyclopedia of Genes and Genomes (KEGG) pathway analysis was performed to identify which key signalling pathways the identified biomarkers involved.

The DAVID analysis tool included all biomarkers of Set 1 (51 of targeted analysis) (Refs 4, 14-16, 25-33, 34-40, 41-51) and Set 2 (31 of genome-wide analysis) (Refs 52-58, 59), respectively.

## Results

### Gene-targeted DNA methylation biomarkers for liquid biopsy

Of 31 publications on targeted BC biomarker research, 47% included data from all BC stages, 19% from nonmetastatic (Stages 0–III) BC, and 6% from metastatic Stage IV disease. In 25% of the studies, staging was not specified. More than half of the studies were performed on plasma biosamples (55%), and 45% were performed on serum. The predominant study methods were quantitative methylation-specific PCR and methylation-specific PCR, used in 62.5% and 25% of the studies. DNA extraction in 34% of the studies was performed using a QIAamp DNA Blood Mini Kit (Qiagen), 22% was performed using a QIAamp Circulating Nucleic Acid Kit (Qiagen), and only one study used the standard phenol-chloroform-ethanol method (3%).

Analysis of 31 selected research articles on methylation biomarkers in BC liquid biopsies revealed 51 analysed biomarkers representative of BC. The most studied of all 51 biomarkers were *RASSF1* methylation, described in 13 studies, and *ESR1* methylation, described in 7 different studies. *APC* and *RARB* gene methylation were analysed in six and five studies, respectively, and the methylation of the *ATM*, *FOXA1*, *MLH1*, *ITIH5*, *NBPF1*, *CDKN2A* and *PTEN* biomarkers was investigated in two studies (Supplementary Table S1).

In these studies, the highest sensitivity and specificity measures for detecting BC were identified at least in one study for *SMAD4*, *PTEN*, *RARB*, *APC* and *DAPK1* gene methylation (Table 1). The sensitivity of these biomarkers was quite high, and the specificity for all of them reached 100% in at least one study.

**Table 1.** The BC biomarkers’ highest specificity and sensitivity values were reported in individual studies

Biomarker	Sensitivity (%) Value^a^ (range^b^)	Specificity (%) Value^a^ (range^b^)	References
*SMAD4*	100	100	(Ref. 15)
*PTEN*	96.4 (96.4–100)	100 (94–100)	(Refs 15, 25)
*RARB*	95.6 (13.3–95.6)	100 (93.6–100)	(Refs 26, 27, 28, 29, 30)
*APC*	93.4 (17–93.4)	100 (94.2–100)	(Refs 26, 27, 29, 31, 32, 33)
*DAPK1*	88.5	100	(Ref. 60)
*RASSF1*	75 (7–75)	96 (74.1–100)	(Refs 27, 28, 29, 30, 31, 32, 33, 34, 35, 36, 37, 60)
*ESR1*	57 (14–100)	99 (10–100)	(Refs 14, 33, 38, 39, 40)

a The highest value of biomarker’s sensitivity and specificity out of all reviewed studies.

b The range of biomarker’s sensitivity and specificity in all reviewed studies.

*RASSF1* (Ras Association Domain Family Member 1) methylation is one of the most frequently analysed alterations in BC with relatively high (74%–100%) specificity, albeit with a wide range of sensitivity values (7%–75%) (Table 1). RASSF1 protein is involved in cell cycle control, apoptosis regulation and microtubule stabilisation. It functions as an inhibitor of mitosis that stops the cell cycle at metaphase, which is essential for the correct alignment of chromosomes at the metaphase plate (Ref. 61). Along with other gene candidates (*APC*, *CCND2*, *FOXA1*, *PSAT1* and *SCGB3A1*), *RASSF1* can enhance BC detection accuracy by up to 94% (Ref. 32). *RASSF1* methylation, which is specific to BC, has an additional advantage as a tool for monitoring the efficacy of neoadjuvant therapy (Ref. 62). In addition, it is predictive of poor overall survival (OS) and disease-free survival (Refs 63, 64). The hypermethylation of *RASSF1* was found to be associated with hormone receptor-positive (HR-positive) status, as the methylation of *RASSF1* is known to be associated with hormone regulation processes (Refs 32, 33). Moreover, node-positive BC patients exhibit greater *RASSF1* methylation levels than node-negative patients; thus, *RASSF1* methylation is a biomarker associated with disease progression (Ref. 31).

Oestrogen receptor 1 gene (*ESR1)* methylation is one of the most analysed biomarkers in BC liquid biopsy. The sensitivity of the biomarker varied between 14% and 100%, and the specificity was found to be 10%–100% (Supplementary Table S1). The most promising diagnostic result was detected by analysing the methylation rate of the *ESR1* (promoter ER3), which showed 57.5% sensitivity and 99% specificity (Ref. 39). Bos et al. revealed the opposite result, with 100% sensitivity and only 10% specificity for the *ESR1* methylation rate (Ref. 40).

*ESR1* encodes an oestrogen receptor (ER) and ligand-dependent transcription factor that forms homo or heterodimers with *ESR2* and has many functions both in reproductive and nonreproductive tissues (Refs 39, 40). The *ESR1* gene contains multiple promoters and eight exons, leading to transcription product diversity (Ref. 65). While hypermethylation of *ESR1* is associated with BC progression (Refs 38, 39), it is not easy to unify *ESR1* gene methylation results, as different studies analyse different promoter areas using different methylation analysis methods.

Although increased ER expression is found in approximately 70% of all BC cases, and these patients commonly receive endocrine therapy, it has been shown that some patients are resistant to treatment. Methylation of *ESR1* is associated with BC transition from ER+ to ER−, leading to anti-oestrogen treatment resistance and disease progression (Ref. 66).

Although *BRCA1* (BRCA1 DNA repair associated) inactivation in BC is usually associated with mutations, methylation of the gene promoter is associated with transcriptional inactivation of *BRCA1* and is a second hit for mutation carriers (Ref. 67). *BRCA1* methylation is found in 10%–15% of all sporadic BC patients, resulting in complete gene silencing and loss of function (Ref. 67). Loss of BRCA1 in BC cells influences the transformation of luminal progenitor cells to a basal-like BC phenotype (Ref. 68). BRCA1 is essential for cellular regulation, including apoptosis and genome stability maintenance via DNA repair. BC cells without functional *BRCA1* gene (mutated or epigenetically silenced) lose the ability to repair DNA double-strand breaks via a homologous repair pathway. Therefore, double-strand break-inducing therapies, such as platinum-based chemotherapy, result in hypersensitivity to treatment (Ref. 69). Studies in ovarian cancer and BC cell lines revealed a similar mechanism of methylated *BRCA1* association with poly (ADP-ribose) polymerase inhibitor, where *BRCA1* hypermethylation results in an enhanced response to treatment (Refs 70, 71, 72).

The *SMAD4* (SMAD family member 4) biomarker analysed in liquid biopsy showed the highest accuracy, with 100% sensitivity and specificity for BC (Ref. 15). This biomarker also had prognostic value, while *SMAD4* methylation was significantly associated with cancer progression (tumour stage, higher grade and lymph node involvement) (Ref. 15). It also exhibited an association with HR-positive BC subtypes (Ref. 15). Moreover, *SMAD4* cfDNA methylation correlated with and was superior to clinically used carcinoembryonic antigen (CEA) and cancer antigen 15.3 (CA15.3) biomarkers (Refs 15, 25). While methylated *SMAD4* may serve as a biomarker with high sensitivity and specificity for early BC detection, along with methylated *PTEN* biomarkers, both act as tumour invasiveness and distant metastasis-associated factors (Ref. 15).

The family of SMAD proteins is responsible for transducing signals within the cell and is involved in numerous signalling pathways. Although SMAD4 loss alone is not linked to carcinogenesis, it interferes with the transforming growth factor beta (TGF-β) and bone morphogenic protein pathways, which can lead to transcriptional activation or inhibition of targeted genes. Additionally, TGF-β/SMAD4 is involved in the DNA damage response and repair by controlling the transcriptional activity of essential genes involved in these processes (Ref. 73). Li et al. reported that SMAD4 is associated with apoptosis in the early stages of ERα-positive BC; therefore, the loss of SMAD4 can induce uncontrolled cell growth due to alterations in cell cycle arrest and apoptosis (Ref. 74).

Phosphatase and tensin homolog (PTEN) methylation in BC liquid biopsy samples showed 96.4% sensitivity and 100% specificity and was a more potent diagnostic tool than CEA and CA15.3. Furthermore, methylated *PTEN* correlated with OS (Ref. 25). *PTEN* is a tumour suppressor gene involved in translation, cell cycle and apoptotic processes. *PTEN* suppresses apoptosis and increases cell survival by negatively regulating the AKT kinase pathway (Ref. 75). Moreover, *PTEN* is involved in DNA repair processes and is essential for BC signalling pathways. The downregulation of *PTEN* leads to the development of malignant mammary stem/progenitor cells through increased signalling within the AKT/GSK-3β/Wnt/β-catenin pathway; moreover, the loss of *PTEN* results in resistance to trastuzumab therapy and poor OS (Ref. 76).

*RARB* (Retinoic Acid Receptor Beta) gene methylation is one of the most frequently analysed epigenetic biomarkers in various cancers (Refs 26, 27, 28, 29). The RARB is a nuclear receptor and a member of the Retinoic Acid Receptor (RAR) class (Ref. 77). It is a transcription initiator activated by a ligand – a physiologically active form of vitamin A (retinoic acid). The primary function of RARB is to control epithelial cell proliferation and haematopoiesis. RARB is essential for signal transduction pathways, cell division and differentiation processes (Ref. 77).

In all studies analysing BC liquid biopsy samples, *RARB* methylation was defined as a diagnostic biomarker with the highest sensitivity (95.6%) and 100% specificity (range 12%–95.6% and 94%–100%, respectively) for detecting BC (Table 1). Swellam et al. defined *RARB* gene methylation as a more robust diagnostic tool than the traditional tumour markers CEA and CA15.3, which are helpful not only for early BC detection but also for early clinical stage, low grade and TNBC definition (90% sensitivity and 100% specificity) (Ref. 26). Kim et al., analysing a set of biomarkers (*SCGB3A1*, *RARB*, *RASSF1* and *TWIST1*), reported *RARB* gene methylation in BC patient serum with a sensitivity of 86.6% and a specificity of 93.6%. Adding to this analysis, a second gene, *RASSF1*, improved the sensitivity to 94.1% and achieved an area under the curve (AUC) of 0.979, although the specificity was lower (88.8%). Nevertheless, the author proposed that these two gene panels are suitable for early and metastatic BC diagnosis (Ref. 30).

*APC* (APC Regulator of WNT Signalling Pathway) is another frequently analysed methylation biomarker specific to colon cancer that is often hypermethylated in BC specimens with high specificity (94.2%–100%) and various ranges of sensitivity (17%–93.45%) (Table 1). A review of studies analysing *APC* hypermethylation highlighted *APC* as a prognostic biomarker associated with advanced tumour stage (Ref. 29), disease progression (Ref. 33) and metastasis (Ref. 31), as this gene is a crucial cell adhesion-regulating factor (Ref. 78). While *APC* acts as a component of the Wnt/β-catenin signalling pathway, the methylation of *APC* dysregulates the signalling pathway and increases resistance to chemotherapeutic agents (Ref. 79). Another *APC* study demonstrated that loss of *APC* function via methylation or mutation acts as an accelerant for *ABCB1* gene expression gain, and as a result, cells became resistant to doxorubicin (Refs 80, 81).

The *DAPK1* (Death-associated protein kinase 1) gene encodes calcium- and calmodulin-dependent serine/threonine kinase involved in cell cycle regulation, autophagy, apoptosis, oxidative stress and metastatic processes. *DAPK1* is essential for regulating AKT kinase, which is involved in many response pathways that induce metastasis: cell spreading, activation of proliferation, inhibition of apoptosis, regulation of p53 and angiogenesis (Ref. 82). A high sensitivity (88%) and 100% specificity of *DAPK1* gene hypermethylation were shown in one BC cfDNA methylation study (Ref. 33). *DAPK1* and *RARB* gene methylation were defined as diagnostic biomarkers, but both showed significant associations with menopausal status (Refs 29, 60).

### DNA methylome profiling in liquid biopsy

In selected publications on liquid biopsy, seven methylome analyses were performed using next-generation sequencing (NGS)-based methods, and one used the EPIC-array method. Three of the eight studies used whole-genome bisulphite sequencing (WGBS) to analyse the whole BC genome. These studies revealed 60,035 differentially methylated regions (DMRs) (Supplementary Table S2). Two studies separated DMRs into hyper and hypomethylated regions, with hypomethylated DMRs comprising the central part of the analysed regions (89% *vs.* 11%) (Supplementary Table S2). Genome-wide hypomethylation is known to be associated with metastatic BC. In contrast, the early stages of the disease show a hypermethylation profile, indicating a change in the methylation pattern during BC progression (Refs 83, 84). Genome-wide methylation research revealed 31 BC biomarkers (Supplementary Table S2).

Widschwendter et al. used a reduced representation bisulphite sequencing method to perform ultradeep bisulphite sequencing for primary and metastatic BC to monitor DNA methylation change before and after chemotherapy (Ref. 52). Ten methylated CpG regions showed the best results and were subsequently optimised to five areas, which covered the DNA methylation marker EFC#93. The hypermethylation of EFC#93 in BC patient serum was a vital marker for both poor relapse-free survival and OS (hazard ratio 5.973). More than 70% of patients who were EFC#93 and CTC-positive relapsed within 5 years, indicating the prognostic potential of this biomarker. Moreover, compared with healthy controls, the methylation-based discrimination of the EFC#93 region in primary and metastatic BC patients showed AUC values of 0.850 and 0.845, respectively. The sensitivity and specificity of this biomarker reached 60.9% and 92%, respectively, when controls were compared to the total group of BC patients. Furthermore, EFC#93 methylation was detected in 43% of women 3–6 months and 25% of them 6–12 months before BC diagnosis with a lethal outcome with 88% specificity and 33.9% sensitivity (fourfold higher than that of nonfatal BC). These results confirm DNA methylation biomarkers’ tremendous prognostic potential and open possibilities for treatment individualisation (Ref. 52).

Using WGBS data from the two BC cohorts, Liu et al. reported the use of cfMETH (a predictive score based on cfDNA methylation in each sample and computed using a random classifier) combined with diagnostic imaging tools (mammography and ultrasound) to develop diagnostic tests with high sensitivity, specificity and accuracy (95.2%, 78.4%, and 86.8%, respectively, taking the average of both cohorts) for diagnosing BC (Ref. 53). The AUC values of the cfMETH predictive score in the discovery and validation cohorts were 0.89 and 0.81, respectively. When analysing 10 optimal hypo-DMRs distinguishing malignant and benign plasma samples, four genes associated with these DMRs were reported as DNA methylation biomarkers for BC (*RYR2, RYR3, GABRB3* and *DCDC2C*). When comparing WGBS data of the BC genome with those of healthy controls, a hypomethylation pattern at the genome-wide level was found (Ref. 53).

Another WGBS study revealed predominant hypomethylation in BC cfDNA samples (64.5%) and associated 146 genes with differentially methylated CpGs (DMCpGs) and 204 with hypo-DMCpGs (Ref. 54). Methylation levels in 13 CpGs were comparable in BC tissue and cfDNA and significantly different between cancerous specimens and noncancerous controls (both in tissues and cfDNA). Thirteen CpGs were described as diagnostic biomarkers and associated with nine genes (Supplementary Table S2). Three of the 13 CpGs were further analysed in an additional cohort. They showed high sensitivity and specificity for diagnosing BC (69.4%–83.7% and 85.7%–88.6%, respectively), indicating that these sites could serve as biomarkers for early-stage BC diagnosis (Ref. 54).

Rodriguez-Casanova et al. used the EPIC array method and detected 28,799 DMCpGs in cfDNA from nine metastatic luminal B patients compared to healthy controls (Ref. 55). As in previous studies, hypomethylated DMCpGs were dominant (92%) and were found in low-density CpG areas (open sea) and outside the promoters. Hypermethylation was predominant in CpG islands and promoters, and 1,467 DMCpGs were generated to differentiate BC from control samples. Thirty-four DMCpGs corresponded to 24 genes associated with the Wnt signalling pathway. Analysis of *WNT1* gene hypermethylation revealed a difference in patients with the luminal B subtype of BC *versus* healthy controls, with an AUC of 0.86, a sensitivity of 78%, and a specificity of 100%. Moreover, researchers are claiming, that *WNT1* gene hypermethylation serves not only as a diagnostic BC biomarker but also as a tool for metastasis monitoring (Ref. 55).

Yang et al. used the reduced representative methylome profiling (RRMP) method. They identified CpGs in the promoter (75.2%) and CpG island areas (81.9%), with the ability to distinguish BC patients from controls, including patients with benign breast lesions and an AUC of 0.85 (Ref. 56). By performing an RRMP analysis on BC lines, the group identified an association between H3K4me3 and hypermethylation, suggesting that this method could be used for histone modification analysis (Ref. 56).

SPOT-MAS (Screening for the Presence Of Tumor by Methylation And Size) refers to a multi-modal liquid biopsy assay that analyzes cell-free DNA in the blood—examining methylation patterns, fragment size profiles, copy-number variations, and end motifs—to detect tumors. Two groups of researchers, including 462 BC patients, performed two SPOT-MAS research analyses (Refs 57, 58). In both studies, the cfDNA concentration was higher in cancerous specimens, and short DNA fragments (<150 bp) were associated with BC, indicating that the cfDNA of cancer patients was more fragmented than that of healthy participants. Both studies analysed EMs and found that different 4-mers increased (CA** and GG**) and decreased (CG** and A***) in cancer specimens (Refs 57, 58).

Among all the variables included in the SPOT-MAS assay, EM and genome-wide methylation had the best results for cancer detection, with sensitivities of 58.3% and 49.3%, respectively, and specificities >95% in two BC cohorts (Ref. 57). The detection rate of BC was the lowest among the five studied cancer types, which could be explained by the low level of cfDNA shedding and molecular subtype heterogeneity (Ref. 85). By measuring the importance scores of different cfDNA features, the best results for all five cancer types were obtained for BC, with the highest values of 0.87 and 0.78 in the two cohorts, respectively (Ref. 57).

Pham et al. performed SPOT-MAS analysis on 239 nonmetastatic BC cfDNA specimens (Ref. 58). Interestingly, as Liu J’s results (Ref. 53) showed increased methylation instability in the TNBC and HER2 BC subtypes, Pham’s work revealed a similar tendency, with the TNBC and luminal B-HER2 subtypes exhibiting a significant proportion of hypermethylation (Ref. 58). Moreover, four regions of DMR covering the *SOX17*, *RASSF1* and *OTX2* genes with significant hypermethylation rates distinguished BC patients from healthy individuals (Ref. 58).

Using WGBS, Liu et al. analysed samples from 16 patients with hormone receptor-positive BC (Ref. 59). The group’s main goal was to analyse ctDNA methylation in patients before and after disease relapse while using exemestane (EXE) therapy. The group identified 79 differential methylation density (MD) regions, which covered 175 genes, and 70 differential methylation ratios (MRs), which covered 223 genes, associated with resistance to EXE. Moreover, the MD and MR regions included seven DMRs in common, which overlapped with various genes participating in the anti-tumour immune response (*HLA*) or apoptosis and cell cycle regulation (*TRIM42*) (Supplementary Table S2). Interestingly, according to the MD and MR data, regions covering the genes *SUCLG2-DT1*, *CLSTN2*, *CLSTN2-AS1*, *TRIM42* and *ANO4* were associated with prognosis for progression-free survival (PFS) after EXE treatment. When predicting EXE resistance, changes in MD and MR in the region covering HLA class II α chain paralogous family genes (*HLA-DRA*, *HLA-DRB1*, *HLA-DRB5* and *HLA-DRB6*) were detected, and a greater methylation rate of this region was associated with shorter PFS (Ref. 59).

### The function and importance of selected biomarkers

The DAVID bioinformatics tool (Refs 23, 24) included all 51 targeted hypermethylation biomarkers (Set 1) for further analysis. Concerning the association of Set 1 with human disease, 42 of the 51 biomarkers (82.4%) were associated by GAD disease classification to oncological (54.9%), metabolic (49.0%), neurological (41.2%), cardiovascular system (41.2%) or immune (31.4%) disorders. The same analysis used genome-wide methylation data of 31 BC characteristic biomarkers (Set 2). Analysis revealed that 74.2% of Set 2 biomarkers were associated with metabolic (54.8%), neurological (48.4%), cardiovascular (38.7%), immune (35.5%) and haematological (29.0%) diseases.

**Figure 3.**
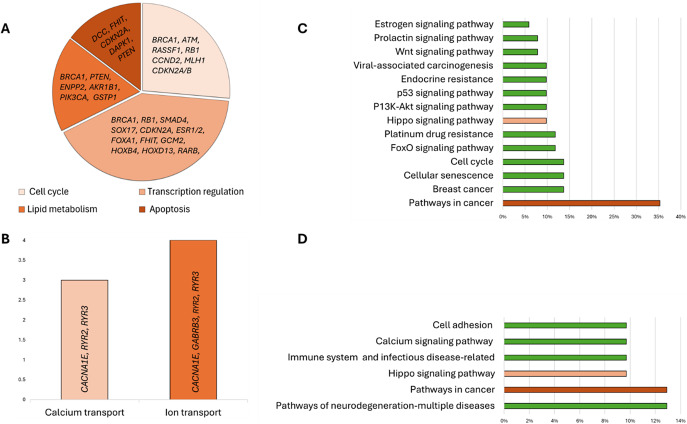
Distribution of biomarkers by their associations with the main cellular processes (**A** and **B**) and signalling pathways (**C** and **D**). Set 1 (**A** and **C**) and Set 2 (**B** and **D**) biomarkers’ analysis.

In further analysis, Set 1 biomarkers were associated with cellular processes (74.5%). The biomarkers were involved in the cell cycle (17.6%), lipid metabolism (11.8%) and apoptosis (9.8%), while most biomarkers were essential for transcription-associated processes (27.5%) (Figure 3A). Moreover, seven biomarkers identified in the Set 2 analysis were related to two cellular transport-associated processes (Figure 3B).

The participation of the targeted biomarkers in various signalling pathways was analysed using KEGG pathway analysis, which revealed that 68.6% of the analysed Set 1 genes participated in different signalling pathways, with most of the genes (35.3%) involved in cancer-related pathways (Figure 3C). Seven genes were associated with BC (*APC, BRCA1, RB1, ESR1, ESR2, PTEN* and *PIK3CA*); among these genes, only *RB1* participates in cell cycle control, while the other six participate in different signalling pathways. The PI3K–Akt, Prolactin and oestrogen signalling pathways had the most common genes associated with BC, while the Hippo, Wnt and p53 signalling pathways shared one gene in common with BC (Table 2). Genome-wide methylation-associated biomarkers were involved in the pathways of neurodegeneration-multiple diseases (13%), cancer-related pathways (*DCC, RASSF1, WNT1, CTNNA2*; 13%), and Hippo, calcium signalling, cell adhesion and immune system-related pathways (all 10%) (Figure 3D).

**Table 2.** Signalling pathways and associated hypermethylation biomarkers

Signalling pathway	PAB (%)	Hypermethylation biomarkers
BC	15	*APC, BRCA1, RB1, ESR1, ESR2, PTEN, PIK3CA*
Cell cycle	15	*ATM, **RB1**, SMAD4, CCND2, CDKN2A, CDKN2B, SFN*
FoxO	13	*ATM, SMAD4, CDKN2B, **PTEN**, **PIK3CA***
Hippo	10	***APC**, RASSF1, SMAD4, CDH1, CCND2*
P13K–Akt	10	***BRCA1**, COL6A2, CCND2, **PTEN**, **PIK3CA***
p53	10	*ATM, CCND2, CDKN2A, **PTEN**, SFN*
Wnt	8	***APC**, SMAD4, SOX17, CCND2*
Prolactin	8	*CCND2, **ESR1**, **ESR2**, **PIK3CA***
Oestrogen	6	***ESR1**, **ESR2**, **PIK3CA***

%: the percentage of the Set 1 genes associated with indicated signalling pathways; BC, breast cancer; PAB: pathway-associated biomarkers.

*Note*: The biomarkers in bold overlap with BC-associated biomarkers.

When data from methylome analysis with targeted biomarkers were compared, two BC-associated overlapping genes (*SOX17* and *RASSF1*) were found. The main pathways of methylome biomarkers were associated with calcium signalling (*CACNA1E, RYR2, RYR3*), cancer pathways (*DCC, RASSF1*, *WNT1, CTNNA2*), multiple neurodegenerative diseases (*WNT1, DNAI1, RYR2, RYR3*), immune system disorders and cell adhesion (*HLA-DRA, HLA-DRB1, HLA-DRB5*).

## Discussion

### Current liquid biopsy utility in clinical practice

Liquid-biopsy assays analysing CTCs in BC have been in use in the clinic for nearly 20 years (Ref. 86). However, the only commercially available DNA methylation-based test for BC is the Therascreen^®^ (QIAGEN) test (Ref. 87). The test is based on *PITX2* (paired-like homeodomain transcription factor 2) methylation analysis in tumour tissue and is used to predict responses to anthracycline-based chemotherapy in ER-positive HER2-negative BC (Ref. 88). The test uses a Qiagen real-time PCR kit and runs in a Rotor-Gene Q MDx thermal cycler (QIAGEN) enabling the detection of three CpG sites in the *PITX2* gene. DNA methylation of *PITX2* predicts a poor response to chemotherapy in patients with lymph node-positive BC and the risk of distant metastasis in patients with node-negative BC (Refs 89, 90). Unfortunately, there is still no alternative test based on liquid biopsy.

### Diagnostic and prognostic value of methylation biomarkers in liquid biopsies for BC

Our review analysis shows that certain methylation biomarkers present strong diagnostic and prognostic potential in BC, especially in more advanced disease settings. This review confirms that DNA methylation biomarkers analysed in liquid biopsies have strong potential to improve early BC detection and assist in the personalisation of treatment. Methylation analysis of genes such as *SMAD4*, *PTEN, APC* and *RARB* is suitable for early diagnosis and discrimination of malignant cases from non-cancerous controls with high sensitivity (>90%) and specificity (100%) and markedly outperforms clinically used protein biomarkers CEA and CA15.3. Some of the reviewed biomarkers have strong prognostic potential, allowing earlier determination of disease progression (*APC, RASSF1, ESR1, TMEM240*), association with BC stage (*GBP2, RASSF1, APC, PTEN, SMAD4*), lymph node metastasis (*GBP2, ESR1, PTEN*) or poor differentiation grade (*ESR1, RASSF1, SMAD4*). Moreover, *ESR1, PTEN* and *TMEM240* methylation in liquid biopsy was associated with poor PFS and OS (Supplementary Table S1). In addition to specific genes, hypermethylation of the EFC#93 region, identified in an epigenome-wide study, was shown to predict the RFS and OS of patients with fatal BC 1 year before the event occurred (Ref. 52). Additionally, methylation of BC-related genes could provide essential insights into treatment response prediction, allowing clinicians to personalise treatment options according to the needs of individual patients, as shown by research investigating *APC*, *BRCA1*, *PTEN*, *TMEM240, ENPP2* and *ESR1* methylation profiles (Supplementary Table S1).

When analysing genes strongly associated with relevant carcinogenic pathways, several genes with essential functions were highlighted, including *ESR1, PTEN, APC, SMAD4* and *RASSF1.* The genes *RASSF1*, *SOX17* and *DCC* showed diagnostic potential in gene-targeted and epigenome-wide studies (Supplementary Tables S1 and S2). Moreover, the *RASSF1* and *SOX17* genes were overlapping in targeted and genome-wide analysis of our review analysis. Both genes are implicated in the regulation of cellular processes such as proliferation, differentiation and progression, and their hypermethylation has been associated with BC (Refs 91, 92). However, no overlap between the genomic regions selected for validation in various genome-wide studies was observed, pointing to the need for further technology development for bisulphite conversion and NGS of cfDNA fragments in liquid biopsy.

**Figure 4.**
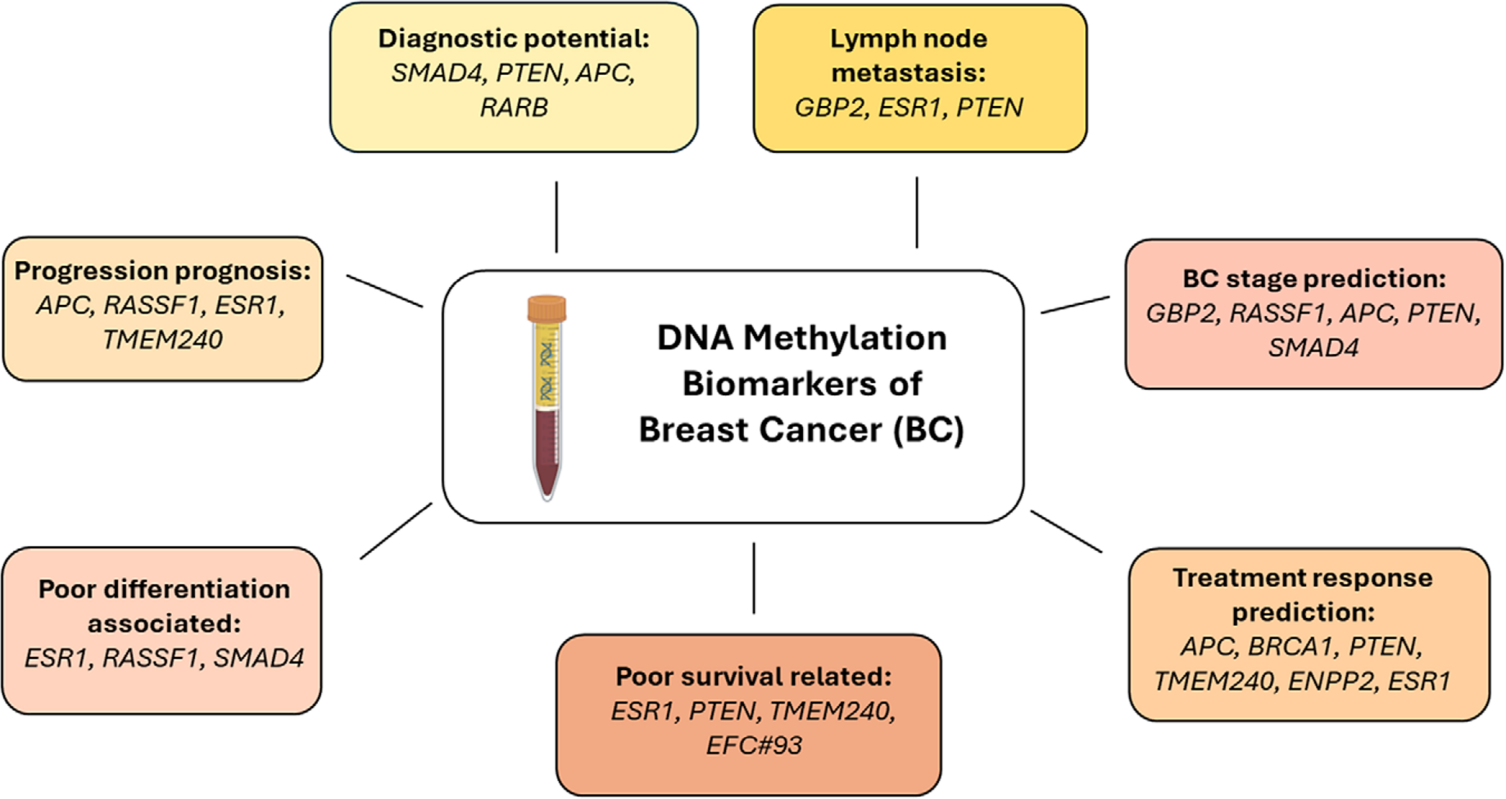
Breast cancer liquid biopsy biomarkers with the greatest diagnostic, prognostic or predictive significance.

Based on this review, we can expect that testing for *RASSF1*, *RARB, SMAD4*, *TMEM240*, *ESR1*, *PTEN, APC* genes, and EFC#93 region hypermethylation can provide the most value due to their high diagnostic and prognostic sensitivity (Figure 4). Moreover, this set of genes is essential in the cell cycle, apoptosis, transcriptional regulation and cell migration processes and participates in various signalling pathways the dysregulation of which is crucial for cancer formation (Ref. 93). It indicates that these biomarkers should be validated in independent BC cohorts.

Single-gene or gene set analysis by real-time or digital PCR techniques allows faster translation of laboratory-derived tests into *in vitro* diagnostics (IVD) tools. Such IVD tests are already available for colon (Cologuard^®^ stool-DNA-based test; Epi proColon^®^ 2.0 test; EarlyTect^®^ CRC assay), cervical (Cervi-M^®^ assay), lung (Epi proLung BL Reflex Assay^®^), glioblastoma (Therascreen MGMT Pyro Kit), bladder (Bladder EpiCheck^®^) and other tumours (Refs 94, 95). However, strict EU regulations and differences in national rules on clinical use and reimbursement policies remain the major issues for the wider application of such tests in the clinic.

## Conclusions

Individualised consideration of DNA methylation profiles of tumour in conjunction with other clinical features would allow clinicians to adopt personalised patient management plans, facilitate early diagnosis and accurate prognosis, tailor treatment and monitoring strategies to each patient, and implement personalised medicine. While additional evidence is required before liquid biopsy-based DNA methylation tests can be implemented in the clinic, we can cautiously assume that they will have a place in BC management.

## Supporting information

Sadzeviciene et al. supplementary material Sadzeviciene et al. supplementary material

## Data Availability

The datasets supporting the conclusions of this article are included within the article and in Supplementary Tables S1 and S2.

## Abbreviations

AUC: area under the curve
BC: breast cancer
cfDNA: cell-free DNA
cfMETH: a predictive score of cfDNA methylation
CTC: circulating tumour cell
ctDNA: circulating tumour DNA
DMCpGs: differentially methylated CpGs
DMRs: differentially methylated regions
EM: end motif
EXE: exemestane
GAD: Gene–Disease Association Database
IVD: *in vitro* diagnostics
KEGG: Kyoto Encyclopedia of Genes and Genomes
MD: methylation density
miRNA: microRNA
MR: methylation ratios
NGS: next-generation sequencing
OS: overall survival
PFS: progression-free survival
ncRNA: non-coding RNA
RRMP: reduced representative methylome profiling
SPOT-MAS: screening for the presence of tumours by DNA methylation and size
TGF-β: transforming growth factor beta
TNBC: triple-negative breast cancer
WGBS: whole-genome bisulphite sequencing

## Supplementary material

The supplementary material for this article can be found at http://doi.org/10.1017/erm.2025.10008.
