# Supplementary material for: Liquid Biopsy-Based DNA Methylation Biomarkers for Precision Medicine in Breast Cancer
Source: Expert Rev Mol Med. 2025 Jun 17;27:e20. doi: 10.1017/erm.2025.10008 (PMC12285340; doi:10.1017/erm.2025.10008)
Supplement: Sadzeviciene et al. supplementary material [file S1462399425100082sup001.docx]

**Table S1.** **All analyzed BC biomarkers with clinical-pathological, research methods, analysis result data, and biomarker value.**

| **Ref.** | **Year** | **Cohort** | **Adjuvant Treatment** | **Sample** | **Stage** | **DNA extraction method** | **Method** | **Biomarker** | **AUC** | **Sensitivity, %** | | **Specificity, %** | | **Marker type** |
| --- | --- | --- | --- | --- | --- | --- | --- | --- | --- | --- | --- | --- | --- | --- |
| [29] | 2006 | 47 BC and 38 HC | No | Plasma | I-IV | ph/chl | qMSP | ***APC*** |  | 17 | 62 | 100 | 87 | Dgn. |
|  |  |  |  |  |  |  |  | ***GSTP1*** |  | 26 |  | 100 |  |  |
|  |  |  |  |  |  |  |  | ***RASSF1**** |  | 32 |  | 92 |  |  |
|  |  |  |  |  |  |  |  | ***RARB**** |  | 26 |  | 95 |  |  |
| [35] | 2006 | 50 BC and 14 HC | No | Plasma |  | QDBM | qMSP | ***RASSF1**** |  | 26 | 36 | 100 | 100 | Dgn. |
|  |  |  |  |  |  |  |  | ***ATM*** |  | 14 |  | 100 |  |  |
| [14] | 2008 | 106 BC and 74 HC | Mixed | Serum | I-IV | QDBM | qMSP | ***ESR1*** | 0.69 | 75 | 81 | 53 | 88 | Dgn. |
|  |  |  |  |  |  |  |  | ***SFN**** |  | 65 |  | 62 |  |  |
| [33] | 2009 | 79 BC and 19 HC | Mixed | Serum | NA | ZZR | qMSP | ***APC*** |  | 29 | 53 | 95 | 84 | Dgn/Prg. (progression) |
|  |  |  |  |  |  |  |  | ***RASSF1**** |  | 35 |  | 100 |  |  |
|  |  |  |  |  |  |  |  | ***ESR1*** |  | 20 |  | 89.5 |  |  |
| [30] | 2010 | 119 BC and 125 HC | No | Serum | I-III | IGBDE | qMSP | ***SCGB3A1**** | 0.58 | 30.3 | 98.3 | 100 | 81.6 | Dgn. |
|  |  |  |  |  |  |  |  | ***RARB**** | 0.93 | 86.6 |  | 93.6 |  |  |
|  |  |  |  |  |  |  |  | ***RASSF1**** | 0.6 | 32.8 |  | 95.2 |  |  |
|  |  |  |  |  |  |  |  | ***TWIST1**** | 0.66 | 54.6 |  | 92 |  |  |
| [4] | 2010 | 26 BC and 12 HC | No | Serum | I-IV | QDBM | MSP | ***DAPK1*** |  | 88.5 | 96.2 | 100 | 92 | Dgn. |
|  |  |  |  |  |  |  |  | ***RASSF1**** |  | 69.2 |  | 92 |  | Dgn/Prg (grade) |
| [28] | 2012 | 101 BC and 87 HC | NA | Serum | I-IV | DPK | OS-MSP | ***GSTP1*** |  | 4 | 22 | 98 | 94 | Dgn. |
|  |  |  |  |  |  |  |  | ***RASSF1**** |  | 7 |  | 100 |  |  |
|  |  |  |  |  |  |  |  | ***RARB**** |  | 12 |  | 95 |  |  |
| [85] | 2013 | 123 BC and 37 HC | NA | Plasma | I-III | HPVNA | MSP | ***CST6*** | NA | 39.8 |  | 100 | NA | Dgn. |
| [36] | 2013 | 138 BC and 135 HC | No | Serum | I-IV | QCNA | qMSP | ***ITIH5*** | 0.69 | 13.8 | 67 | 94.8 | 69 | Dgn. |
|  |  |  |  |  |  |  |  | ***DKK3*** |  | 29.7 |  | 98.5 |  |  |
|  |  |  |  |  |  |  |  | ***RASSF1**** |  | 47.1 |  | 74.1 |  |  |
| [86] | 2013 | 114 BC and 49 HC | Mixed | Plasma | I-IV | HPVNA | qMSP | ***SOX17*** |  | 34.5; 40.7 |  | 98 |  | Dgn. |
| [40] | 2014 | 120 BC and 100 BenC | No | Plasma | 0-III | QDBM | MSP | ***ESR1 prom ER3*** | NA | 57.5 |  | 99 |  | Dgn/Prg. (grade; lymph node) |
|  |  |  |  |  |  |  |  | ***ESR1 prom ER4*** |  | 21.7 |  | 100 |  |  |
|  |  |  |  |  |  |  |  | ***ESR1 prom ER5*** |  | 55.8 |  | 96 |  |  |
| [37] | 2014 | 24 BC and 28 HC (training); 33 BC and 27 HC (test) | Yes | Serum | IV | QMinE | qMSP | ***AKR1B1*** | 0.95; 0.99 | 63; 42 | 92; 91 | 100; 100 | 96; 100 | Dgn. |
|  |  |  |  |  |  |  |  | ***ARHGEF7*** |  | 29; 18 |  | 100; 100 |  |  |
|  |  |  |  |  |  |  |  | ***COL6A2*** |  | 37; 36 |  | 96; 100 |  |  |
|  |  |  |  |  |  |  |  | ***GPX7*** |  | 21; 30 |  | 100; 100 |  |  |
|  |  |  |  |  |  |  |  | ***HOXB4*** |  | 46; 36 |  | 100; 100 |  |  |
|  |  |  |  |  |  |  |  | ***RASGRF2*** |  | 50; 45 |  | 98; 100 |  |  |
|  |  |  |  |  |  |  |  | ***RASSF1**** |  | 75; 67 |  | 96; 100 |  |  |
|  |  |  |  |  |  |  |  | ***TM6SF1*** |  | 42; 36 |  | 93; 100 |  |  |
|  |  |  |  |  |  |  |  | ***TMEFF2*** |  | 8; 18 |  | 100; 100 |  |  |
|  |  |  |  |  |  |  |  | ***H3C3**** |  | 50; 55 |  | 100; 93 |  |  |
| [87] | 2015 | 36 BC and 30 HC |  | Serum |  | CDEK | pyro | ***FHIT*** |  | 64.2 |  | 64.7 |  | NA |
|  |  |  |  |  |  |  |  | ***BRCA1*** |  | 10 |  | 98.3 |  |  |
| [26] | 2015 | 121 BC and 66 HC | No | Serum |  | QDBM | MSP | ***RARB**** |  | 95.6 |  | 100 |  | Dgn. |
|  |  |  |  |  |  |  |  | ***APC*** |  | 93.4 |  | 100 |  |  |
| [38] | 2016 | 268 BC and 245 HC | NA | Serum | NA | QCNA | qMSP | ***MLH1**** | 0.73 | 27.9 | 79.6 | 85.7 | 72.4 | Dgn. |
|  |  |  |  |  |  |  |  | ***RASSF1**** |  | 17.2 |  | 89.7 |  |  |
|  |  |  |  |  |  |  |  | ***CDKN2A**** |  | 22.4 |  | 83.3 |  |  |
|  |  |  |  |  |  |  |  | ***PCDHGB7*** |  | 55.6 |  | 52.7 |  |  |
|  |  |  |  |  |  |  |  | ***SFN*** |  | 73.5 |  | 41.6 |  |  |
|  |  |  |  |  |  |  |  | ***HOXD13*** |  | 13.8 |  | 97.6 |  |  |
| [88] | 2016 | 132 BC and 51 HC | NA | Plasma | I-IV | QCNA | qMSP | ***SEPTIN9**** |  | 26.5 |  | 100 |  | Dgn/Prg. (grade; HR+; non-pCR) |
| [27] | 2017 | 165 BC and 67 HC | NA | Plasma | I-IV | QCNA | MSP | ***CDKN2A**** | NA | 13.3 | NA | NA | 61.2 | NA |
|  |  |  |  |  |  |  |  | ***CDKN2B**** |  | 7.2 |  | NA |  |  |
|  |  |  |  |  |  |  |  | ***CDKN2A**** |  | 11.5 |  | NA |  |  |
|  |  |  |  |  |  |  |  | ***RB1*** |  | 8.4 |  | NA |  |  |
|  |  |  |  |  |  |  |  | ***APC*** |  | 37.5 |  | NA |  |  |
|  |  |  |  |  |  |  |  | ***DCC*** |  | 21.8 |  | NA |  |  |
|  |  |  |  |  |  |  |  | ***CDH1**** |  | 1.8 |  | NA |  |  |
|  |  |  |  |  |  |  |  | ***TIMP3*** |  | 3.6 |  | NA |  |  |
|  |  |  |  |  |  |  |  | ***ATM*** |  | 4.2 |  | NA |  |  |
|  |  |  |  |  |  |  |  | ***RASSF1**** |  | 34.5 |  | NA |  |  |
|  |  |  |  |  |  |  |  | ***BRCA1*** |  | 3.6 |  | NA |  |  |
|  |  |  |  |  |  |  |  | ***MLH1**** |  | 2.4 |  | NA |  |  |
|  |  |  |  |  |  |  |  | ***VHL*** |  | 0.6 |  | NA |  |  |
|  |  |  |  |  |  |  |  | ***RARB**** |  | 13.3 |  | NA |  |  |
| [89] | 2018 | 52 BC and 30 HC | No | Plasma | I-III | QDBM | MSP | ***NBPF1*** | NA | 67.1 |  | 59.1 |  | Dgn. |
| [90] | 2018 | 18 BC and 19 HC | NA | Plasma | I-IV | MMAX | qMSP | ***CCND2*** |  | 44.4 |  | 84.2 |  | Dgn. |
| [32] | 2018 | 44 BC and 39 HC | No | Plasma | I-III | QMinE | qMSP | ***APC*** | 0.63 | 27.27 | 81.82 | 94.87 | 76.9 | Dgn. |
|  |  |  |  |  |  |  |  | ***FOXA1*** | 0.73 | 68.18 |  | 82.05 |  |  |
|  |  |  |  |  |  |  |  | ***RASSF1**** | 0.57 | 13.64 |  | 100 |  |  |
| [31] | 2018 | 108 BC and 103 HC | No | Plasma | 0-IV | QMinE | qMSP | ***APC*** |  | 32.41 |  | 94.2 |  | Dgn |
|  |  |  |  |  |  |  |  | ***FOXA1*** |  | 38.9 |  | 79.6 |  |  |
|  |  |  |  |  |  |  |  | ***RASSF1*** |  | 19.4 |  | 100 |  |  |
|  |  |  |  |  |  |  |  | ***SCGB3A1*** |  | 21.3 |  | 92.2 |  |  |
| [91] | 2019 | 31 BC and 219 BenC | No | Serum | I | QCNA | pyro | ***SPAG6*** | 0.67 | 44 | 63 | 82 | 79 | Dgn |
|  |  |  |  |  |  |  |  | ***ITIH5*** | 0.79 | 74 |  | 85 |  |  |
| [39] | 2020 | 49 luminal-like MBC | Yes | Plasma | IV | Qsym | MS-ddPCR | ***ESR1*** | NA | 14 |  |  |  | Dgn/Prg. (poor PFS, metastasis) |
|  |  |  |  |  |  |  |  | ***PIK3CA*** |  | 23 |  |  |  |  |
| [92] | 2020 | 84 BC and 75 HC | No | Plasma | II-IV | QDBM | MSP | ***GBP2*** | NA | 90.50 |  | 94.7 |  | Dgn/Prg. (stage; lymph node) |
| [25] | 2021 | 112 BC and 25 HC | No | Serum | I-IV | QDBM | qMSP | ***PTEN*** | 0.99 | 96.4 |  | 100 |  | Dgn/Prg. (poor PFS and OS) |
| [15] | 2021 | 80 BC and 30 HC | No | Serum | I-V | QDBM | qMSP | ***PTEN*** | 0.99 | 100 |  | 94 |  | Dgn/Prg. (stage; grade; lymph node; HR+; HER2) |
|  |  |  |  |  |  |  |  | ***SMAD4*** | 0.85 | 100 |  | 100 |  |  |
| [93] | 2021 | 57 BC and 134 HC | NA | Plasma | 0-IV | QDBM | qMSP | ***CCDC181*** | 0.95 | 65.9 | 97.7 | 77.4 | 85.5 | Dgn. |
|  |  |  |  |  |  |  |  | ***GCM2*** |  | 61.4 |  | 75.7 |  |  |
|  |  |  |  |  |  |  |  | ***ITPRIPL1*** |  | 66.7 |  | 74.1 |  |  |
| [94] | 2022 | 49 BC and 49 BenC |  | Serum | NA | QCNA | qMSP | ***PCDHB15*** | 0.59 | 40.8 |  | 77.6 |  | Dgn. |
| [95] | 2022 | 86 BC and 46 HC | No | Plasma | I-IV | QDBM | qMSP | ***ENPP2*** |  | 72.1 |  | 54.4 |  | Dgn/Prg. (TR) |
| [41] | 2022 | #1. 31 BC and 20 HC  #2. 18 BC and 10 HC | Yes | Plasma |  | #1. Max | qMSP | ***ESR1*** |  | 100 |  | 10 |  | age-related |
|  |  |  | Yes |  |  | #2. QCNA |  |  |  | 94.4 |  | 30 |  |  |
| [16] | 2022 | 61 BC | Yes | Plasma |  | MMAX | qMSP | ***TMEM240*** |  | 87.5 |  | 93.1 |  | Prg. (Progression; TR) |

**Abbreviations:** AUC – area under curve; BC – breast cancer; BenC – benign controls; CDEK – cfDNA extraction kit (Omega); Dgn – diagnostic; DPK – DNA purification kit (Qiagen); HC – healthy controls; HPVNA – High Pure Viral Nucleic Acid Kit (Roche Diagnostics); HR+ - hormone receptor positive; IGBDE – I-genomic Blood DNA Extraction Mini Kit (iNtRON Biotechnology); Lum – luminal; Max – Maxwell (MX) RSC ccfDNA Plasma Kit (Promega); MBC – metastatic breast cancer; MMAX – MagMAX Cell-Free DNA Isolation Kit (TFS); MS-ddPCR – methylation-specific droplet digital PCR; MSP – methylation-specific PCR; NA – not available; pCR – pathologic complete response; prom – promoter; OS – overall survival; OS-MSP – one-step methylation-specific PCR; PFS – progression free survival; ph/chl – phenol/chloroform; pyro – pyrosequencing; Prg – prognostic; QCNA – QIAamp Circulating Nucleic Acid Kit (Qiagen); QDBM – QIAamp DNA Blood Mini Kit (Qiagen); QMinE – QIAamp MinElute ccfDNA (Qiagen); Qsym – QIAsymphony SP PAXgene Blood ccfDNA Kit (Qiagen); TR – treatment response; ZZR – ZR Serum DNA Kit (Zymo Research).

* Gene names clarified according to the “HUGO Gene Nomenclature Committee” (https://www.genenames.org/).

**Table S2. Genome-wide methylation profile analysis of BC cfDNA.**

| **Ref.** | **Year** | **Cohort** | **Adjuvant Treatment** | **Sample** | **Stage** | **DNA extractionmethod** | **Method** | **Methylated regions no.** | **Methylated regions location** | **Biomarker** |
| --- | --- | --- | --- | --- | --- | --- | --- | --- | --- | --- |
| [67] | 2021 | 77 BC and 77 BenC | NA | plasma | I-III | QCNA | WGBS | 51,962 hypo-DMR and 5613 hyper-DMR | NA | *RYR2, RYR3, GABRB3, DCDC2C* |
| [74] | 2020 | 16 HR+ BC | yes | plasma | I-IV | MMAX | WGBS | 79 DMD and 70 DMR | all 23 | *LINC00623, LINC00869, SUCLG2-DT*, PTPN20, CLSTN2, CLSTN2-AS1, TRIM42, HLA-DRA, HLA-DRB5, HLA-DRB6, HLA-DRB1, ANO4, FAM74A1* |
| [68] | 2021 | 10 BC and 10 HC | no | plasma | 0-IV | MCD | WGBS | 1,453 DMCpG (937 hypo-DMCpG) | NA | *FABP9, LCE1A, CACNA1E, CPEB4, DNAI1, DLGAP2, BCAS3, CTNNA2, DCC, GALNT8* |
| [69] | 2022 | 9 BC (LB) and 5 HC | NA | plasma | IV | QCNA | EPIC array | 28,799 DMCpG | all 23 | *WNT1* |
| [66] | 2017 | 419 BC before and 415 BC after chemo | yes | serum | I-IV | NA | RRBS | NA | chr 1-4, 8, 9, 11-12, 16, 17, 19, X | EFC#93 |
| [70] | 2024 | 29 BC and 27 NBC | no | plasma | I-III | PCDE | RRMP | NA | NA | NA |
| [71] | 2023 | 223 BC and 1550 HC | NA | plasma | I-IIIA | MMAX | SPOT-MAS | NA | chr 1-22 | NA |
| [72] | 2023 | 72 BC and 97 HC | NA | plasma | I-IIIA | MMAX | SPOT-MAS | NA | 22 chr | *SOX17*, RASSF1*, OTX2** |

**Abbreviations:** BC – breast cancer; BenC – benign controls; CNA – DNA copy number; chr – chromosome; DMCpG – differentially methylated CpGs; DMD – differential methylation density; DMR – differentially methylated regions; HC – healthy controls; LB – luminal B; MCD – Mapure Circulating DNA Kit (Magen); MMAX – MagMAX Cell-Free DNA Isolation Kit (TFS); NA – not available; QCNA – QIAamp Circulating Nucleic Acid Kit (Qiagen); PCDE – Plasma Cell-Free DNA Extraction Kit (Concert); RRBS – reduced representation bisulfite sequencing; RRMP – reduced representative methylome profiling; SPOT-MAS – screening for the presence of tumors by DNA methylation and size; WGBS – whole-genome bisulfite sequencing.

* Gene names clarified according to the “HUGO Gene Nomenclature Committee” (https://www.genenames.org/).
